# Supplementary material for: Teleworking and Musculoskeletal Disorders: A Systematic Review
Source: Int J Environ Res Public Health. 2023 Mar 11;20(6):4973. doi: 10.3390/ijerph20064973 (PMC10049341; doi:10.3390/ijerph20064973)
Supplement: Supplementary file 1 [file ijerph-20-04973-s001.zip › ijerph-2219768-supplementary.pdf]

Supplemental Material S1: Strings used for searching the selected databases

PubMed

("telework"[Title/Abstract] OR "teleworking*"[Title/Abstract] OR "teleworker"[Title/Abstract] OR "telecommut*"[Title/Abstract] OR "remote work*"[Title/Abstract] OR "telecommuting"[Title/Abstract] OR "remote working" [Title/Abstract] OR "work from home"[Title/Abstract] OR "work at home"[Title/Abstract] OR "working from home"[Title/Abstract] OR "working at home"[Title/Abstract] OR "Home-Based Work"[Title/Abstract] OR "home-based working"[Title/abstract] OR "telehomework"[Title/Abstract] OR "home-working"[Title/abstract] OR "home working"[Title/Abstract] OR "homeworking"[Title/Abstract] OR "home-office" [Title/Abstract] OR "flexible work"[Title/Abstract] OR "virtual office"[Title/Abstract] OR "virtual work"[Title/Abstract] OR "home office"[Title/Abstract] OR "e-work"[Title/Abstract] OR "e-workers"[Title/Abstract] OR "e-working"[Title/Abstract])

AND

("musculoskeletal pain" [Title/Abstract] OR "musculoskeletal disorder"[Title/Abstract] OR "musculoskeletal risk"[Title/Abstract] OR "musculoskeletal disease"[Title/Abstract] OR "neck pain"[Title/Abstract] OR "neck injury"[Title/Abstract] OR "cervico-brachial"[Title/Abstract] OR "cervical pain"[Title/Abstract] OR "cervicalgia"[Title/Abstract] OR "tension neck"[Title/Abstract] OR "back pain"[Title/Abstract] OR "back injury"[Title/Abstract] OR "shoulder pain"[Title/Abstract] OR "shoulder injury"[Title/Abstract] OR "shoulder tendonitis"[Title/Abstract] OR "shoulder tendinitis"[Title/Abstract] OR "subacromial"[Title/Abstract] OR "elbow pain"[Title/Abstract] OR "elbow injury"[Title/Abstract] OR "elbow tendonitis"[Title/Abstract] OR "epicondyl"[Title/Abstract] OR "elbow tendinitis"[Title/Abstract] OR "epicondylitis"[Title/Abstract] OR "wrist pain"[Title/Abstract] OR "wrist tendonitis"[Title/Abstract] OR "wrist tendinitis"[Title/Abstract] OR "wrist injury"[Title/Abstract] OR "arm pain"[Title/Abstract] OR "arm injury"[Title/Abstract] OR "carpal tunnel syndrome"[Title/Abstract] OR "carpal tunnel"[Title/Abstract] OR "work related injury"[Title/Abstract] OR "work related musculoskeletal disorder"[Title/Abstract] OR "WMSD"[Title/Abstract] OR "MSD"[Title/Abstract] OR "occupational overuse"[Title/Abstract] OR "neck tension"[Title/Abstract] OR "muscular function"[Title/Abstract] OR "muscle pain"[Title/Abstract] OR "limb disorder" [Title/Abstract] OR ("musculoskeletal" [Title/Abstract] AND "epidemiology" [Title/Abstract]) OR ("musculoskeletal" [Title/Abstract] AND "etiology" [Title/Abstract]))

AND (("1987/01/01"[Date - Entry] : "3000"[Date - Entry]))

AND (English[Language] OR French[Language])

Web of Science

("telework" OR “teleworking*" OR "teleworker" OR "telecommut*" OR "remote work*" OR “telecommuting" OR “remote working” OR "work from home" OR "work at home" OR "working from home" OR "working at home" OR "Home-Based Work" OR “home-based working" OR "telehomework" OR "home-working" OR "home working" OR "homeworking" OR “home-office” OR "flexible work" OR "virtual office" OR "virtual work" OR "home office" OR "e-work" OR "e-workers" OR "e-working")

AND ("musculoskeletal pain" OR "musculoskeletal disorder" OR "musculoskeletal risk" OR "musculoskeletal disease" OR "neck pain" OR "neck injury" OR "cervico-brachial" OR "cervical pain" OR "cervicalgia" OR "tension neck" OR "back pain" OR "back injury" OR "shoulder pain" OR "shoulder injury" OR "shoulder tendonitis" OR "shoulder tendinitis" OR "subacromial" OR "elbow

pain" OR "elbow injury" OR "elbow tendonitis" OR "epicondyl" OR "elbow tendinitis" OR "epicondylitis" OR "wrist pain" OR "wrist tendonitis" OR "wrist tendinitis" OR "wrist injury" OR "arm pain" OR "arm injury" OR "carpal tunnel syndrome" OR "carpal tunnel" OR "work related injury" OR "work related musculoskeletal disorder" OR "WMSD" OR "MSD" OR "occupational overuse" OR "neck tension" OR "muscular function" OR "muscle pain" OR "limb disorder" OR (“musculoskeletal” AND “epidemiology”) OR (“musculoskeletal” AND “etiology”)) (Abstract)

and English OR French (Language)

and 1987-2022 (Year Published)

+

(("telework" OR “teleworking*" OR "teleworkers" OR "telecommut*" OR "remote work*" OR “telecommuting" OR “remote working” OR "work from home" OR "work at home" OR "working from home" OR "working at home" OR "Home-Based Work" OR “home-based working" OR "telehomework" OR "home-working" OR "home working" OR "hotworking" OR “home-office” OR "flexible work" OR "virtual office" OR "virtual work" OR "home office" OR "e-work" OR "e-workers" OR "e-working")

AND ("musculoskeletal pain" OR "musculoskeletal disorder" OR "musculoskeletal risk" OR "musculoskeletal disease" OR "neck pain" OR "neck injury" OR "cervico-brachial" OR "cervical pain" OR "cervicalgie" OR "tension neck" OR "back pain" OR "back injury" OR "shoulder pain" OR "shoulder injury" OR "shoulder tendonitis" OR "shoulder tendinitis" OR "subacromial" OR "elbow pain" OR "elbow injury" OR "elbow tendonitis" OR "epicondyle" OR "elbow tendinitis" OR "epicondylitis" OR "wrist pain" OR "wrist tendonitis" OR "wrist tendinitis" OR "wrist injury" OR "arm pain" OR "arm injury" OR "carpal tunnel syndrome" OR "carpal tunnel" OR "work related injury" OR "work related musculoskeletal disorder" OR "wmsn" OR "MSD" OR "occupational overuse" OR "neck tension" OR "muscular function" OR "muscle pain" OR "limb disorder" OR (“musculoskeletal” AND “epidemiology”) OR (“musculoskeletal” AND “etiology”))) (Title)

and English OR French (Language)

and 1987-2022 (Year Published)

Embase

('telework':ab OR 'teleworking*':ab OR 'teleworker':ab OR 'telecommut*':ab OR 'remote work*':ab OR 'telecommuting':ab OR 'remote working':ab OR 'work from home':ab OR 'work at home':ab OR 'working from home':ab OR 'working at home':ab OR 'home-based work':ab OR 'home-based working':ab OR 'telehomework':ab OR 'home-working':ab OR 'home working':ab OR 'homeworking':ab OR 'home-office':ab OR 'flexible work':ab OR 'virtual office':ab OR 'virtual work':ab OR 'home office':ab OR 'e-work':ab OR 'e-workers':ab OR 'e-working':ab)

AND ('musculoskeletal pain':ab OR 'musculoskeletal disorder':ab OR 'musculoskeletal risk':ab OR 'musculoskeletal disease':ab OR 'neck pain':ab OR 'neck injury':ab OR 'cervico-brachial':ab OR 'cervical pain':ab OR 'cervicalgia':ab OR 'tension neck':ab OR 'back pain':ab OR 'back injury':ab OR 'shoulder pain':ab OR 'shoulder injury':ab OR 'shoulder tendonitis':ab OR 'shoulder tendinitis':ab OR 'subacromial':ab OR 'elbow pain':ab OR 'elbow injury':ab OR 'elbow tendonitis':ab OR 'epicondyl':ab OR 'elbow tendinitis':ab OR 'epicondylitis':ab OR 'wrist pain':ab OR 'wrist tendonitis':ab OR 'wrist

tendinitis':ab OR 'wrist injury':ab OR 'arm pain':ab OR 'arm injury':ab OR 'carpal tunnel syndrome':ab OR 'carpal tunnel':ab OR 'work related injury':ab OR 'work related musculoskeletal disorder':ab OR 'wmsd':ab OR 'msd':ab OR 'occupational overuse':ab OR 'neck tension':ab OR 'muscular function':ab OR 'muscle pain':ab OR 'limb disorder':ab OR ('musculoskeletal':ab AND 'epidemiology':ab) OR ('musculoskeletal':ab AND 'etiology':ab))

AND ([english]/lim OR [french]/lim)

AND [1987-2022]/py

+

('telework':ti OR 'teleworking*':ti OR 'teleworker':ti OR 'telecommut*':ti OR 'remote work*':ti OR 'telecommuting':ti OR 'remote working':ti OR 'work from home':ti OR 'work at home':ti OR 'working from home':ti OR 'working at home':ti OR 'home-based work':ti OR 'home-based working':ti OR 'telehomework':ti OR 'home-working':ti OR 'home working':ti OR 'homeworking':ti OR 'home-office':ti OR 'flexible work':ti OR 'virtual office':ti OR 'virtual work':ti OR 'home office':ti OR 'e-work':ti OR 'e-workers':ti OR 'e-working':ti)

AND ('musculoskeletal pain':ti OR 'musculoskeletal disorder':ti OR 'musculoskeletal risk':ti OR 'musculoskeletal disease':ti OR 'neck pain':ti OR 'neck injury':ti OR 'cervico-brachial':ti OR 'cervical pain':ti OR 'cervicalgia':ti OR 'tension neck':ti OR 'back pain':ti OR 'back injury':ti OR 'shoulder pain':ti OR 'shoulder injury':ti OR 'shoulder tendonitis':ti OR 'shoulder tendinitis':ti OR 'subacromial':ti OR 'elbow pain':ti OR 'elbow injury':ti OR 'elbow tendonitis':ti OR 'epicondyl':ti OR 'elbow tendinitis':ti OR 'epicondylitis':ti OR 'wrist pain':ti OR 'wrist tendonitis':ti OR 'wrist tendinitis':ti OR 'wrist injury':ti OR 'arm pain':ti OR 'arm injury':ti OR 'carpal tunnel syndrome':ti OR 'carpal tunnel':ti OR 'work related injury':ti OR 'work related musculoskeletal disorder':ti OR 'wmsd':ti OR 'msd':ti OR 'occupational overuse':ti OR 'neck tension':ti OR 'muscular function':ti OR 'muscle pain':ti OR 'limb disorder':ti OR ('musculoskeletal':ti AND 'epidemiology':ti) OR ('musculoskeletal':ti AND 'etiology':ti))

AND ([english]/lim OR [french]/lim)

AND [1987-2022]/py

EBSCO databases

"telework" OR “teleworking*" OR "teleworker" OR "telecommut*" OR "remote work*" OR “telecommuting" OR “remote working” OR "work from home" OR "work at home" OR "working from home" OR "working at home" OR "Home-Based Work" OR “home-based working" OR "telehomework" OR "home-working" OR "home working" OR "homeworking" OR “home-office” OR "flexible work" OR "virtual office" OR "virtual work" OR "home office" OR "e-work" OR "e-workers" OR "e-working"

AND

"musculoskeletal pain" OR "musculoskeletal disorder" OR "musculoskeletal risk" OR "musculoskeletal disease" OR "neck pain" OR "neck injury" OR "cervico-brachial" OR "cervical pain" OR "cervicalgia" OR "tension neck" OR "back pain" OR "back injury" OR "shoulder pain" OR "shoulder injury" OR "shoulder tendonitis" OR "shoulder tendinitis" OR "subacromial" OR "elbow pain" OR "elbow injury" OR "elbow tendonitis" OR "epicondyl" OR "elbow tendinitis" OR

"epicondylitis" OR "wrist pain" OR "wrist tendonitis" OR "wrist tendinitis" OR "wrist injury" OR "arm pain" OR "arm injury" OR "carpal tunnel syndrome" OR "carpal tunnel" OR "work related injury" OR "work related musculoskeletal disorder" OR "WMSD" OR "MSD" OR "occupational overuse" OR "neck tension" OR "muscular function" OR "muscle pain" OR "limb disorder" OR ("musculoskeletal" AND "epidemiology") OR ("musculoskeletal" AND "etiology")

Language: English (filter)

Years: 1995 à 2022 (filter)

+

"telework" OR "teleworking*" OR "teleworker" OR "telecommut*" OR "remote work*" OR "telecommuting" OR "remote working" OR "work from home" OR "work at home" OR "working from home" OR "working at home" OR "Home-Based Work" OR "home-based working" OR "telehomework" OR "home-working" OR "home working" OR "homeworking" OR "home-office" OR "flexible work" OR "virtual office" OR "virtual work" OR "home office" OR "e-work" OR "e-workers" OR "e-working"

AND

"musculoskeletal pain" OR "musculoskeletal disorder" OR "musculoskeletal risk" OR "musculoskeletal disease" OR "neck pain" OR "neck injury" OR "cervico-brachial" OR "cervical pain" OR "cervicalgia" OR "tension neck" OR "back pain" OR "back injury" OR "shoulder pain" OR "shoulder injury" OR "shoulder tendonitis" OR "shoulder tendinitis" OR "subacromial" OR "elbow pain" OR "elbow injury" OR "elbow tendonitis" OR "epicondyl" OR "elbow tendinitis" OR "epicondylitis" OR "wrist pain" OR "wrist tendonitis" OR "wrist tendinitis" OR "wrist injury" OR "arm pain" OR "arm injury" OR "carpal tunnel syndrome" OR "carpal tunnel" OR "work related injury" OR "work related musculoskeletal disorder" OR "WMSD" OR "MSD" OR "occupational overuse" OR "neck tension" OR "muscular function" OR "muscle pain" OR "limb disorder" OR ("musculoskeletal" AND "epidemiology") OR ("musculoskeletal" AND "etiology")

Language: English (filter)

Years: 1995 à 2022 (filter)

Cairn

(teleworking OR teletravail OR "travail à distance" "travail à domicile")

(sante OR health OR musculo-squelettique OR musculosquelettique OR musculoskeletal)

Google Scholar

allintitle: ("work from home" OR telecommuting OR telework OR "remote work" OR "e work") AND (musculoskeletal)

Supplementary material S2: Full summary of data extracted from selected studies

Author	Country	During COVID	Population	Recruitment and data collection	Exclusion	Number included	Dates of inclusion /follow-up	Definition of MSK outcome	Confounding	Results	Conclusion of authors	Limitations of authors
Aegerter, 2021	Switzerland	Yes	Swiss office workers aged 18–65 years, working more than 25 h per week in sedentary office work, able to communicate in German, in the control cohort between January and April 2020, who answered the COVID-19-related questions in full and were working from home at the time of follow-up	Longitudinal study based on data from an ongoing stepped-wedge cluster randomized controlled trial. Baseline data collected with a 30-min online questionnaire	Severe health conditions such as previous trauma or injuries of the neck, inflammatory disease, any history of cervical spine surgery or if exercise was contraindicated	58	October to December 2019. 4 to 5 months follow-up	NP severity and disability in the last 4 weeks: numeric rating scale and ND index	Fixed effects for workstation ergonomics, working hours at the computer, number of breaks during work, and time	No evidence that ND, number of work breaks, number of hours of computer work changed between pre COVID-19 (WFH). Evidence of a 0.68-point reduction in NP intensity during the lockdown (95% CI 1.35 to 0.00). Possible increase of the effect of number of hours working on a computer and quality of workplace ergonomics on NP intensity. Possible decrease of the effect of number of daily work breaks on ND. Strong evidence of poorer workstation ergonomics at home compared to the office	No evidence that number of breaks, or hours of computer work changed between pre COVID-19 pandemic (office work) and follow-up during lockdown (WHF). Number of hours working on a computer and quality of workplace ergonomics may increase effect on NP intensity, whereas the number of daily work breaks may decrease ND	Population in the public sector, social desirability bias, low follow-up, retrospective report of workstation ergonomics at the office at follow-up, no objective criteria for assessing workstation ergonomics
Argus, 2021	Estonia	Yes	Job described as office work, working with a computer at least 6 hours per day, age 18–60 years.	Recruited by contacting their organization's occupational health and safety officers. Online questionnaire	Chronic neurological, orthopedic, metabolic, or inflammatory conditions, and legally designated disability	161	May to June 2020	Modified NORDIC MSK questionnaire (personalized scale assessing the onset and evolutivity of the pain)	None	There were no statistically significant differences in the prevalence of MSP before and during the COVID-19 lockdown in different body areas and in total.	No significant differences in the prevalence of overall MSK pain before and during the lockdown	Questionnaire-based design, retrospective questions, and absence of data about psychosocial factors and pain intensity
Bailly, 2022	France, Switzerland	Yes	From the multicenter CONFI-LOMB study regrouping patients from hospitals and one private rheumatology: Adult who undergone a consultation for a common chronic LBP between January 1, 2020, and March 17, 2020 (start of the French lockdown)	The physicians contacted eligible patients by phone or sent them a form or an electronic version of the questionnaire after sending an information letter	Suffering from another chronic pain syndrome interfering with daily life activities, having comorbidities limiting the practice of physical ability, poor understanding of the French language and inability to answer a questionnaire	360	May 12 to June 30, 2020	Change in LBP intensity prior to the lockdown and during lockdown assessed by a 7-point Likert scale	None	In bivariate analyses, LBP increased in case of teleworking during lockdown (p=0.069) but not in case of workstation dedicated to teleworking (p=0.249) or equipment adapted to telework (p=0.355)	Teleworking seemed to affect LBP significantly	Cross-sectional design, self-administered and anonymous computerized questionnaire patients from tertiary centers

Deshmukh, 2020	India	Yes	IT professionals working 100% from home for at least a 6 month period	Google form circulated to IT professionals after taking their data from LinkedIn via social media	Professional working partially from home and office	100	May to October 2020	Nordic MSK Questionnaire assessing MSKD	None	Results are difficult to interpret because of lack of details	After quarantine, 30% raise in work related MSK pain in IT professionals	Self-reported data, possibly underestimated their WMSDs to avoid being viewed negatively and small sample size
El Kadri Filho, 2022	Brazil	Yes	Employees of a Regional Labor Court who were teleworking specifically because of the need for social isolation	Invited to participate in the research through an institutional e-mail. Online survey and video calls	Employees on vacation or sick leave, employees already teleworking before March 2020	55	November to December 2020	Nordic MSK questionnaire assessing MSKD	None	Regions with most complaints in the last 6 months and last 7 days were shoulders, neck, and wrists/hands. Posture and job demand exposure assessment was significantly corelated with MSK problems	No relationship between the results of the workstation evaluations with the occurrence of MSK symptoms in teleworking employees	Only workers in the labor judiciary, data collection eight months after the onset of the pandemic, cross-sectional design
Gerding, 2021	USA	Yes	All faculty staff, and administration employed by the University of Cincinnati	Survey sent to participants (no information on type of survey)	None	843	April 14, 2020	Level of discomfort numeric rating scale for several body regions	None	>40% employees reported moderate to severe discomfort levels in the eyes/neck/head, upper back/shoulders, and lower back regions. Prior to COVID-19, 78.5% experienced little to no discomfort while working in their office setting, and 21.5% had moderate to severe discomfort. Increased glare and lack of having contact with the back of the chair increased discomfort for various body areas	Large increases in discomfort levels following the stay-at-home orders were observed	Only staff from one public university, self-reported data on postures and discomfort, retrospective data, and low response rate (10%)
Guler, 2021	Turkey?	Yes	Workers from companies that started working from home after the pandemic and who practice desk work using a computer	Purposive sampling to sample from certain profession. Online survey	Working from home part-time (only on certain days of the week), starting work after the pandemic	194	October to November 2020	Nordic MSK Questionnaire and Visual Analog Scale for work-related pain	Multivariate analysis included independent variables with p-values<0.05 in the univariate analyses	Mean LBP significantly increased during pandemic (WFH) compared to before: 3.14 to 3.56 ($p = 0.03$). LBP was associated with lumbar support before the pandemic, stress level during WFH, general health status, sleep duration and rest quality during WFH in the multivariate regression model	Significant increases were observed in the complaints of LBP of employees due to ergonomic deficiencies	Cross-sectional design, low number of participants, some specific activities (treadmill, exercise bikes, pilates) were not considered separately, no evaluation of COVID infection during WFH and no assessment of income changes during WFH

Houle, 2021	Canada	Yes	Participants aged between 18 and 65 years old and in a full-time telecommuting situation since at least one week prior to enrollment	Recruited via social media platforms (Facebook and University web platforms). Baseline online survey and daily online survey on five consecutive days	Head and/or neck trauma in the past 6 months	162	May to July 2020. 5 days	NP occurrence, intensity (numerical rating scale) and NP assessed by the Neck Bournemouth Questionnaire (NBQ)	Multivariate analysis with a stepwise method	70% reported at least one NP episode during follow-up. No work-related variables were associated with occurrence of NP episode, including presence of home workstation, headset wearing, telecommuting hours and headset wearing hours. Among telecommuters, NP related disability was associated with future NP occurrence	Only NP-related disability was an associated factor of NP occurrence following an acute adaptation to telecommuting	Significant attrition following the initial assessment, impossibility to assess other health complaints, no distinction between headache and NP types, small sample size and no assessment of potential factors
Jain, 2022	India	Yes	University computer users at two Indian universities, over 18 years old, attending regular online classes, performing at least 150-200 min of PA per week	Online survey	No previously diagnosed health issues (neurological, physical, biochemical or inflammation diseases, officially recognized impairment)	40	March to September 2020. 6 months	Corlett and Bishop's body part discomfort scale	None	The region with the highest discomfort on the Corlett and Bishop's scale before intervention were wrist/forearm (8.17+/-1.45), lower back (8.01+/-1.42) and neck (7.40 +/- 2.71)	High occurrence of MSK disorders among computer users in the university. Ergonomic intervention significantly reduced discomfort	Only university students
Knardahl, 2022	Norway	No	All office workers from private and public organizations recruited from a previous project between 2004–2019	Organizational level convenience sampling method. Web-based and paper (15%) surveys	None	Cross-sectional sample: 7861 Prospective sample: 5258	2004 to 2019. 17 to 36 months	Reported NP in the last four weeks with a four-level intensity scale and duration of complaint	Adjusted for working more than regular hours, gender, age, skill level, management responsibility, year of measurement (mixed effects regression)	NP not statistically associated with time working at home (hours): aOR and 95% CI compared to 0 hours: 0 to 2h, 0.93 (0.77–1.12), 2 to 5h, 0.89 (0.71–1.11), 5 to 15, 0.82 (0.63–1.07), more than 15h, 0.65 (0.38–1.11). Availability expectations was associated with NP	Availability expectations, but not hours working at home, was associated with NP	Changes in contents of office jobs between 2004-2020, subjective reports and cross-sectional design
MacLean, 2022	Canada	Yes	Staff, administration and faculty employed by Dalhousie University and primarily working from home with limited on campus access to offices and laboratories since lockdown	Announcements in university newsletters, employment association social media, department level e-mails, and word of mouth. Online survey	None	445	February 2021	Changes in work-related discomfort since WFH (Likert scale) and current discomfort and pain using the Nordic MSK Questionnaire and a numeric rating scale	None	61% reported an increase in MSK pain. Area with most reported at least moderate pain were neck, shoulders, and lower back. Seat height and monitor distance were associated with MSK discomfort or pain, respectively β and 95% CI: 6.0 (3.1, 9.0) and 3.3 (0.5,5.9)	Participants worked in varied environments. Women had higher MSK pain than men, but this effect was mediated by women having worse ergonomic setups at-home. Seat height and monitor distance were associated with greater pain scores	Population may be biased (more administrative, women), selection bias, model could not account for certain aspects of home workstation, self-assessed, anonymous WFH ergonomics and perceived musculoskeletal discomfort and pain

Matsugaki, 2021	Japan	Yes	Population of workers currently in possession of an employment contract who responded that they mainly performed desk work and telecommuted at least once a week	Sampling plan based on geographical area, sex, and office and non-office worker status. Online survey commissioned by a survey company, which has 4.7 million registered monitors	Identifiably false responses	3663	December 22 to December 26, 2020	Reported occurrence of stiff shoulder, of LBP in the past 2 weeks and the average severity of LBP on a numeric rating scale	Adjusted for age, sex, bmi, lifestyle habit (smoking, drinking, PA), number of days with poor mental health (past 30 days), income, educational background, working time, frequency of telecommuting, company size	Telecommuting environment factors (yes/no) associated with LBP were: aOR 95% CI, having enough light on desk 1.43 (1.18-1.73), having enough space to stretch legs 1.30 (1.10-1.54), having a place to concentrate on work 1.38 (1.17-1.64), having appropriate temperature and humidity comfort 1.32 (1.13-1.56), having enough space on the desk 1.19 (1.02-1.39). Using an office desk or chair was no associated with LBP	Telecommuting environment was associated with LBP in home workers during the pandemic. Insufficient desk and foot space, inadequate lighting, uncomfortable temperature and humidity, lack of room/space for work were associated with the prevalence of LBP	History, medication of LBP are unknown, subjective report of home office, lifestyle factors, and working condition, and possibility of a selection bias (more WFH for LBP workers?
Matsugaki, 2022	Japan	Yes	Population of workers currently in possession of an employment contract	Sampling based on geographical area, sex, and office and non-office worker status. Online survey by a survey company, which has 4.7 million registered monitors	Identifiably false responses, not mainly a desk worker, working less than 5 days per week	12 774	December 22 and December 26, 2020	Reported occurrence of stiff shoulder, of LBP in the past 2 weeks and the average severity of LBP on a numeric rating scale	Adjusted for age, gender, bmi, marital status, education, income, lifestyle habit (smoking, alcohol, PA), psychological status, company size	LBP was associated with frequency of teleworking aOR 95% CI compared to reference (Almost never): ≤1 d/w 1.18 (0.99-1.41), 2 to 3d/w 1.27 (1.08-1.50), ≥4 d/w 1.15 (1.01-1.32), p-value of trend = .003. In good telecommuting environment, OR did not increase with telecommuting frequency contrary to poor teleworking environment	Relationship between LBP and work from home differed depending on the quality of the work environment. LBP is associated with WFH when work environment is poor	Subjective assessment of work environment, potential unmeasured confounds, no consideration of duration of workers' telecommuting engagement and cross-sectional design
Minoura, 2021	Japan	Yes	Respondents selected from panelists registered within a Japanese Internet survey agency	Participants recruited using a random sampling method based on age, gender, and region of residence. Online survey	Provided Identifiably responses, non-desk workers, desk workers with LBP before the COVID-19 pandemic, age <18 years or ≥60 years	4227	August to September 2020	Occurrence of LBP in the last month and time of appearance (before/after pandemic)	Adjusted for age, sex, weight, education, marital status, having children, outdoor PA during the COVID-19, psychological distress, smoking, alcohol, comorbidities, employment, income level, working time	LBP was associated with increased WFH (yes vs no) among desk workers during the Covid-19 pandemic: aOR 2.13 (1.52–2.97)	Increased work from home was associated with LBP. Appropriately equipping the work environment of desk workers who start working from home seems to improve productivity and well-being of workers	Cross-sectional design, low response rate, potential selection bias, no information on medical LBP diagnosis and quality of work environment, residual confounders, possible time lag between survey and LBP

Moretti, 2020	Italy	Yes	Population of mobile workers employed as administrative officers that moved to work remotely since the beginning of COVID-19 health emergency	Participants were contacted by phone	None	51	Not detailed	Brief Pain Inventory for assessing LBP and NP	None	Main regions for MSK pain were the low back (41.2%) and the neck (23.5%). Since WFH, NP worsened for 50% participants (N=6), and 38.1% for LBP (N=8)	Most participants reported worsening NP, but no increased of LBP was reported. Results suggest that MSKD related to WFH might reduce job satisfaction	Small sample size, population of a single Italian region, coss-sectional design, some confounders could not be assessed
Muniandy, 2022	Malaysia	Yes	Lecturers and students of UMS who were actively involved in teaching and learning during the pandemic period	Convenience sampling. Online survey via an Internet-based application	Known spinal deformities, history of back pain before the COVID-19 quarantine	842	May 2020	Back pain intensity using a numeric rating scale	None	Among newly diagnosed back pain lumbar region was the most frequent (62.1%), and LBP increased after lockdown. Poor ergonomic sitting was associated with mild LBP: OR CI 95% 2.0 (1.2-3.6)	Significant increase in sitting time and a decrease in PA during lockdown, which contributed to an increase in the frequency of back pain	Population of predominantly people higher formal education, survey online accessibility
Oakman, 2022	Australia	Yes	Participants from across Australia, aged 18 years or older, working from home at least 2 days per week during the period following declaration of the COVID-19 pandemic in Australia, currently living in Australia	Convenience sample. Recruited through an advertisement distributed via the Facebook paid service, and professional networks of the research team (LinkedIn, Facebook)	None	924	September to November 2020	MSK pain frequency using a 5-point Likert scale and intensity using a 3-point Likert scale	None	After the pandemic, WFH increased for most participants (92.9%). Over 70% reported pain or discomfort at the end of their working day, with higher level of neck/shoulder pain and hips/legs/feet pain for females	WFH may impact employees' physical and mental health, and that this impact is likely to be gendered	Potential selection bias due to the geographical and gender sample repartition, cross-sectional design
Prieto-González, 2021	Slovakia	Yes	Pedagogues in Slovakia during the introduction of online classes (January 2021 during COVID-19 pandemic), working in Slovakia in primary, secondary, tertiary, or special needs schools and aged between 18 and 65 years	Random selection method. Recruitment via social media (Facebook, WhatsApp, Messenger)	Male gender, working in kindergartens	782	From 15 January 2021 to 22 January 2021	Pain intensity using a numeric rating scale	None	74.84% reported cervical pain and 67.68% LBP. Number of days of online classes/week was associated with increased pain intensity: 1/w 3.33 (1.17), 2/w 3.17 (1.07), 3/w 3.46 (1.18), 4/w 3.51 (1.08) and 5/w 3.58 (1.01). Teachers not complying with ergonomic recommendations and sitting most of the time had higher level of pain intensity	Highest back pain incidence was reported in the cervical and low back region. Prevalence of back pain reported by secondary school teachers significantly higher than primary school teachers. The most important risk factor for suffering from back pain was lack of PA	Potential selection bias, detailed medical information available

Radulović, 2021	Croatia	Yes	Telecommunications company workers working from home for eight months (from 16 March to 4 December 2020) before joining the study	Online survey distributed by email through company's Safety at Work Unit	None	232	From December 2020 (no precision)	Changes in MSK pain before and after WFH using a 3-point Likert scale	None	Among reported LBP, 39.1% had stronger pain when working at home than in the office. Complaint of more severe pain at home than in the office was correlated with not having an ergonomic chair or office desk, longer working hours at home, disturbance at home and women	There is a higher prevalence of worsened MSK pain in women. Poorer working conditions at home is a risk for MSK pain	No limitations reported
Regmi, 2022	India	Yes	Working population and students across India	Snowball sampling. Online survey through electronic communication sources (email, WhatsApp, and SMS)	None	1302	July 2020	Frequency of work-related MSKD symptoms while using digital using a 4-point Likert scale	None	88% reported work related MSK disorders among which 45% had these symptoms for the first time. MSK symptoms were not statistically associated with WFH (OR 0.61, 95% CI 0.34-1.09), but there were associated with hours of work at home >8h/d (3.06, 1.89-4.96)	High prevalence of MSK symptoms during pandemic. Female gender, greater time on digital devices, less breaks, increased work hours, no changes in home office and no regular PA increased pain	Convenience sampling, students and health care sector population, general health issues and other ocular problems not considered
Rodriguez-Nogueira, 2021	Spain	Yes	Workers at two Spanish universities employed for at least six months, actively teleworking during the confinement period (between march and May 2020)	Convenience sample. Online survey via email	None	472	April and May 2020	Nordic MSK Questionnaire adapted to Spanish	None	There was a decrease of pain reported overall during confinement compared to the previous 12 months (p<0.001)	Increase in frequency of PA and a change between aerobic activities towards strength training and stretching. At the same time, a reduction has been identified in the prevalence of MSK pain	Self-reported stress, pain and PA, cross-sectional design, population of two Spanish universities
Šagát, 2020	Saudi Arabia	Yes	Resident in Saudi Arabia staying in Riyadh before and during the quarantine, aged between 18 and 64 years	Stratified random sampling based on the city districts. Contact through the Riyadh municipality forum groups available on social media. Online survey	Chronic psychological, physiological, or psychosomatic conditions, hospitalized during the pandemic	463	From 10 May 2020 to 17 May 2020	Location and intensity of pain on a numerical scale	None	Among subject who WFH or did distance learning during quarantine, there were more MSK pain reported compared to before (48.3% vs 3.9%). Pain intensity was higher during quarantine than before for subjects WFH or distance learning: mean pain (2.64 vs 1.97). Subjects who telework or had distance learning had statistically higher MSK pain compared to those who did not (2.63 vs 2.27)	Significant increase in LBP intensity and prevalence. Being aged 35 to 49 years old, stress, non-adherence to ergonomic recommendations, prolonged sitting, insufficient practice of PA, and teleworking or distance learning were associated with a higher LBP intensity	Certain measurements not included (inflammatory biomarkers, vitamin D levels), assessment of LBP intensity four weeks after confinement, no inclusion of chronic conditions hospitalized patients

Siqueira, 2020	Brazil	Yes	Brazilian individuals aged between 18 and 59 years	Online survey through social networks	Established diagnosis of dysphonia, undergoing otorhinolaryngological or treatments for voice or larynx, hearing complaints, and/or diagnosis of COVID-19, diseases of the upper airway, worked in home office before the pandemic, participants in the risk range for developing dysphonia	424	June 2020	Frequency (4-point Likert scale) and MSK pain assessed by the "MSK Pain Investigation Questionnaire"	None	Individuals WFH reported statistically higher frequency of pain than those working in the usual workplace. Individuals WFH had increased frequency of pain during pandemic compared to before in the neck (1.23 vs 1.05), shoulders (1.33 vs 1.13), upper back (1.41 vs 1.20)	Individuals who started to WFH during the COVID-19 pandemic showed increased vocal fatigue symptoms and MSK pain in the cervical region during the pandemic	Potential reporting bias on data before pandemic
Tezuka, 2022	Japan	Yes	Full-time workers of the two non-ferrous metal companies, aged 20 years or more, and non-teleworker before the emergency declaration	Web-based self-reported questionnaires	Missing data	917	June to September 2020	Presence of physical symptoms (from leading symptoms in the National Lifestyle Survey of Japan) not due to COVID-19 infection during the emergency	Adjusted for age, sex, bmi, marital status, occupational status, and stiff shoulders before the emergency declaration	Telework frequency was statistically associated with LBP during emergency declaration: compared to 0 days of telework, aOR and 95% CI, 1-2/w 3.83 (1.41–10.36), 3-4/w 6.09 (2.33–15.94), 5 or more/w 5.57 (2.22–14.00)	Higher telework frequency was significantly associated with a higher prevalence of stiff shoulders, eyestrain, and LBP among workers in Japan during the COVID-19 emergency declaration	Cross-sectional design, potential recall bias, potential selection bias due to the low response rate (34.6%), high percentage of men in the sample and survey distribution method
Widianawati, 2020	Indonesia	Yes	WFH workers during the COVID-19 pandemic in Indonesia	Online survey	None	50	July 2020	MSK pain assessed by a numerical scale	None	28% of workers reported complaints of low MSDS with an average pain value of 50.44. Most frequent region for pain was the neck and the lower back	WFH workers have a fairly good design of ergonomic work facility, however, the employees experience moderate MSK complaints. Workers do not experience heavy stress during WFH	No limitations reported

BMI: body mass index, ND: neck disability, NP: neck pain, LBP: LBP: low back pain, WFH: work from home, OR: odds ratio, MSK: musculoskeletal, PA: physical activity
